# Improving the measurement of TMS-assessed voluntary activation in the knee extensors

**DOI:** 10.1371/journal.pone.0216981

**Published:** 2019-06-06

**Authors:** Jeanne Dekerle, Aaron Greenhouse-Tucknott, James G. Wrightson, Lisa Schäfer, Paul Ansdell

**Affiliations:** 1 Fatigue and Exercise Tolerance Laboratory, Centre for Sport and Exercise Science and Medicine (SESAME), University of Brighton, Eastbourne, United Kingdom; 2 Human Performance Laboratory, Faculty of Kinesiology, University of Calgary, Calgary, Canada; 3 Department of Sport, Exercise and Rehabilitation, Faculty of Health and Life Sciences, Northumbria University, Northumbria, United Kingdom; Ritsumeikan University, JAPAN

## Abstract

**Purpose:**

To test the accuracy, validity, reliability and sensitivity of an alternative method for the measure of TMS-assessed voluntary activation (VA_TMS_) in the knee extensors.

**Methods:**

Ten healthy males (24 ± 5 years) completed a neuromuscular assessment protocol before and after a fatiguing isometric exercise: two sets of five contractions (50%, 62.5%, 75%, 87.5%, 100% Maximal Voluntary Contraction; MVC) with superimposed TMS-evoked twitches for calculation of VA_TMS_ using either the first 5 stimulations (1x5C) or all 10 (2x5C). This was performed on two separate occasions (between-day reliability). Accuracy and validity were compared with a routinely used protocol [i.e. 50%, 75%, and 100% of MVC (1x3C) performed three times (3x3C)].

**Results:**

95% confidence interval for estimated resting twitch, a key determinant of VA_TMS_, was similar between 1x5C, 2x5C, and 3x3C but improved by six-fold when compared to 1x3C (*P*<0.05). In a fresh state, potentiated twitch force was unchanged following 1x5C but decreased following 2x5C (*P*<0.05). A recovery was found post-exercise but was smaller for 1x5C compared to 2x5C (*P*<0.05), with no difference between the latter two (*P*>0.05). Absolute reliability was strong enough for both 1x5C and 2x5C to depict a true detectable change in the sample’s VA_TMS_ following the fatiguing exercise (TEM < 3% at rest, <9% post-exercise) but 2x5C was marginally more sensitive to individual’s changes from baseline.

**Conclusion:**

Both 1x5C and 2x5C provide reliable measures of VA_TMS_. However, 1x5C may hold stronger internal validity. Both protocols allow detection of ‘true’ changes in sample’s means but not individual scores following a fatiguing isometric exercise.

## Introduction

The ability of the CNS to activate skeletal muscle (“voluntary activation”, VA) can be assessed using transcranial magnetic stimulation (TMS) of the primary motor cortex (VA_TMS_) using the twitch interpolation technique [1]. The measure of VA was originally applied using supramaximal electrical stimulation of the innervating motor nerve of the investigated muscle(s) with the later use of single pulse TMS allowing for better discernment of the aetiology of potential impairment (i.e. spinal or supraspinal; [1]; corticospinal excitability [2]; inhibitory networks [3]). To calculate VA_TMS_, a TMS evoked superimposed twitch (SIT) during an MVC is expressed relative to an estimated resting twitch (ERT, Eq 1). The ERT is derived from extrapolation of the linear relationship between SIT and voluntary torque in order to obtain a *y*-intercept representing twitch amplitude at rest (*see* [1] for underlying rationale). Since Todd et al., (2003) validated this technique in the elbow flexors, it has been replicated in other muscle groups, including the knee extensors [4, 5].

$${VA}_{TMS}(\%)=\left(1-\frac{SIT}{ERT}\right)\;x\;100$$ (1)

To date, the standard protocol for the evaluation of VA_TMS_ of the knee extensors comprises three levels of contraction intensity (100%, 75%, and 50% of MVC) performed either once [6], twice [7] or three times [8, 9]. Often described vaguely, as highlighted by Todd et al. [10], the method for modelling the SIT-voluntary torque relationship may rely on inclusion of all SIT recorded during the neuromuscular assessment (NMA) leading to the modelling of 3 to 9 points (i.e. 3 contraction levels x 1 repeat = 3 data points [6]; 3 contraction levels x 2 repeats = 6 data points [7]; or 3 contraction levels x 3 repeats = 9 data points [11]) or an average of the repeats for each level of contraction (3 contraction levels x 3 repeats = 3 data points [9]). Some authors also modelled the relationship from each repeat of three levels of contraction (i.e. 3 points) to keep the highest VA_TMS_ for further analysis [3 contraction levels x 1 repeat = 3 data points [12]]. The ‘urgency’ in measuring VA_TMS_ as soon as possible after the end of a fatiguing task seems to have led researchers to opt for the modelling of three- or six-, rather than nine-point relationships. Accordingly, one method based on the performance of only one but longer contraction of decreasing percentages of MVC, has recently been proposed to capture better (i.e. quicker according to the authors) the loss in VA_TMS_ following a 2-min all-out MVC [4]. Accuracy and validity of the above-mentioned NMA protocols routinely used to determine VA_TMS_ have recently been challenged [10, 11].

Accuracy of estimates from a linear regression is highly dependent upon the number of points entered into a model (i.e. both standard error (SE) and associated confidence interval (CI) calculated as **+/-** SE. *t*_α/2_). For example, as presented in Table 1, with a degree of freedom (*df*) of only 1, a three-point SIT-voluntary torque relationship leads to the largest CI for a given ERT and SE-ERT. But this can be reduced considerably when increasing the number of points, even by just one or two SIT measures (Table 1). It is also worthwhile highlighting the small improvement in ERT accuracy beyond five data points.

**Table 1:** 95% CI for ERT and SE-ERT of 35.5 and 3.7 Nm, respectively (Dekerle et al. [11]).

	*t*_α/2_	95% CI (in Nm)	%improvement in 95% CI from the 3-point model
3-point model	12.71	± 47.0	n.a.
4-point model	4.30	± 15.9	66%
5-point model	3.18	± 11.8	75%
6-point model	2.78	± 10.3	78%
9-point model	2.36	± 8.7	81%
10-point model	2.31	± 8.5	82%
30-point model	2.05	± 7.6	84%

Unfortunately, repeated measures of SIT during a NMA protocol can threaten the face validity of the NMA outcomes. The succession of nine voluntary muscular contractions with super-imposed evoked twitches (3 x 50%, 75% and 100% of MVC) reduces maximal force-generating capacity and potentiated twitch force evoked via femoral nerve stimulation (Q_pot_), evidencing the occurrence of peripheral fatigue [11]. Voluntary and electrically-evoked fatiguing contractions may also modulate corticospinal excitability [2, 13, 14] through increased activation of mechano- and chemo-sensitive thin-diameter afferents [3, 13, 15, 16] and excitability of inhibitory networks [13, 14, 17]. While greater rest periods between contractions may offset these deleterious effects when testing a muscle at rest, challenges arise when performing a NMA following a fatiguing exercise as both peripheral and central branches of neuromuscular fatigue display extremely rapid recovery –with substantial restoration observed within minutes [6, 11, 18, 19]. Accordingly, validity of NMA may be threatened by the interaction between a fatigue mechanisms induced by the repeated contractions and stimulations and homeostatic recovery.

Following exercise, a proportional increase in SIT during an MVC, compared to that elicited at rest (i.e. ERT), demonstrates sub-maximal VA and quantifies the development of central fatigue [20]. The absolute reliability, or the measurement error within a sample of stable individuals [21, 22], is therefore of interest when one examines changes in VA_TMS_ after fatiguing exercise. Typical errors of measurement for VA_TMS_ in the knee extensors have been reported to be around 2% in a fresh state [4, 5, 9, 11, 23, 24] and 10% following a fatiguing exercise [11, 24]. However, interpretation of pre- and post-exercise smallest detectable change for both an individual and a sample of individuals raises questions regarding the suitability of current NMA protocols to detect changes in VA_TMS_ following fatiguing exercise [11].

**Fig 1:**
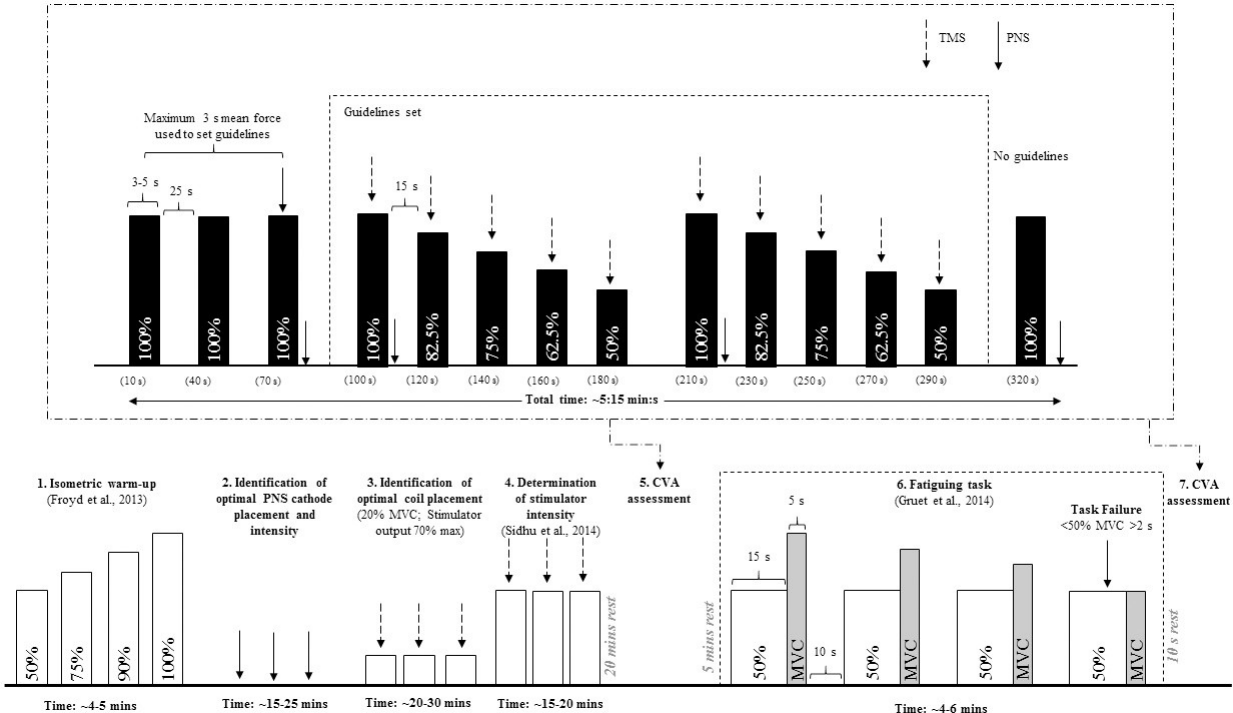
Schematic of the protocol.

Because of the highlighted issues with the current NMA protocols (Dekerle et al. [11]), a new protocol was needed to enhance (1) accuracy, (2) face validity, and (3) absolute reliability and sensitivity of the main NMA outcomes. The present article presents a more robust NMA to allow measurement of VA_TMS_ following fatiguing exercise. Here we propose a five-contraction NMA protocol for the estimation of ERT and subsequent calculation of VA_TMS_ following a fatiguing isometric exercise. In this protocol, five levels of muscular contractions (50%, 62.5%, 75%, 87.5%, and 100% of MVC) were performed twice consecutively (Fig 1).

**Fig 2:**
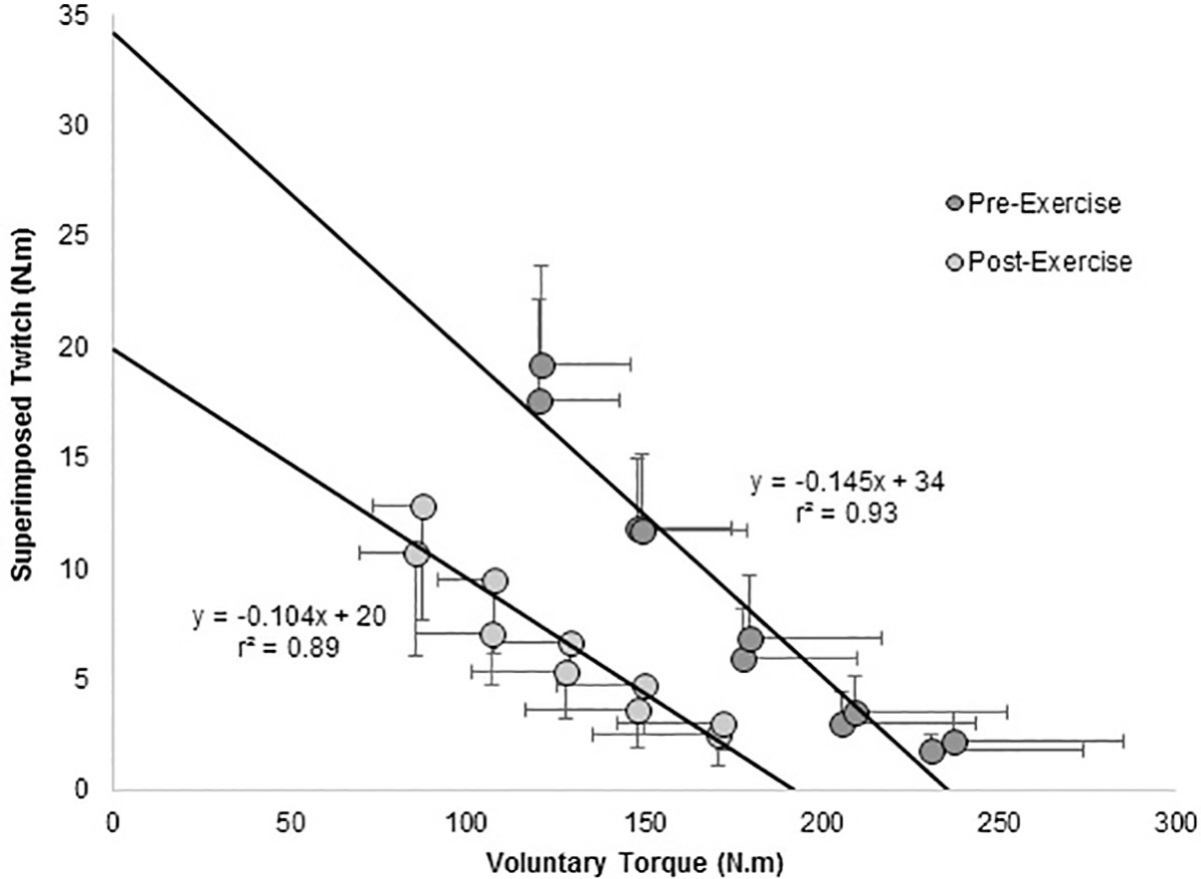
Relationship between voluntary contraction (VC) and superimposed twitch force (SIT) pre- and post-exercise.

The first aim of the present study was to examine goodness of fit (accuracy) of the linear relationships between SIT and voluntary torque (Fig 2). For this first aim, model accuracy was compared with the routinely used three-contraction protocol (1x3C, 3x3C): We hypothesised that 1x5C and 2x5C would reduce confidence intervals in resting twitch estimates (CI-ERT) when compared to 1x3C, while 2x5C and 3x3C would offer the highest level of ERT accuracy. The second aim of the study was to investigate possible threats to internal validity of the 1x5C and 2x5C protocols. Because the performance of multiple contractions may impair neuromuscular function at rest [11], we hypothesised that MVC torque and Q_pot_ would be reduced at rest following 2x5C but not 1x5C. We also examined the converse post-exercise as a two-phase recovery of both peripheral and central fatigue has been reported following high-intensity exercise [6, 18, 19]. We therefore examined whether recovery took place during the NMA performed after the fatiguing exercise. Recovery would be evidenced by increases in Q_pot_, slope, *y*-intercept, and VA_TMS_ following 1x5C and / or 2x5C. Finally, the third aim of the study was to report and compare absolute reliability and sensitivity of the measure of ERT and VA_TMS_ using the 1x5C and 2x5C protocols.

## Methods

### Ethical approval

All experimental procedures were conducted in accordance with the *Declaration of Helsinki*, except for registration in a database, with approval granted by the institute’s research ethics committee (SSCERC 0316, University of Brighton ethics committee). Written informed consent was provided by all volunteers prior to participation.

### Participants

Ten healthy, recreationally active males (mean ± SD; age: 24 ± 5 years) volunteered to participate in the present investigation. Prior to signing consent forms, participants were informed of the purpose of the investigation and completed a screening questionnaire to ensure there were no contraindications to TMS (Rossi et al., 2001). Participants were not taking any medication and had no history of cardiovascular, neurological or musculoskeletal disorders. During the investigation, participants were instructed to refrain from the consumption of both caffeine and alcohol, and the performance of strenuous exercise in the 24 hours preceding each visit.

### Experimental design

Participants visited the laboratories on five separate occasions, with a minimum of 48 hours separating each session (mean experimental duration: 15 ± 5 days). Trials were conducted at the same time of day (± 2 hours) to account for diurnal variations in maximal torque generation and corticospinal excitability [25]. During the first visit, participants were thoroughly familiarised with the performance of MVCs, submaximal contractions, TMS and peripheral nerve stimulation (PNS) before performing a fatiguing single-joint exercise task (*see Fatiguing exercise*). During the remaining four visits, participants completed a pre-fatigue NMA (s*ee NMA protocol*), fatiguing task, and post-fatigue NMA. The fatiguing task consisted of the number of completed contractions recorded during the familiarisation session to match muscular work across trials (*see fatiguing exercise*).

#### NMA protocols

The NMA protocol began with the performance of three brief (3–5 s) MVCs separated by 60 s. Percutaneous electrical stimulation of the femoral nerve was delivered during the final MVC to elicit a superimposed twitch (SIT) as well as 1-2s after contraction ended [26]. The greatest voluntary torque recorded during the three brief maximal contractions was used to set visual guidelines for the individual submaximal torque levels. After 60 s rest, either two sets of five contractions (2x5C protocol: 100, 87.5, 75, 62.5 and 50% MVC) or three sets of three contractions were performed (3x3C protocol: 100, 75, and 50% MVC; *see* details in Dekerle et al. [11]). A femoral stimulation was applied after the 100%MVC to evoke a potentiated twitch torque (Q_pot_). A single TMS pulse was superimposed onto the plateau in force of each contraction. Rest periods of 25 s preceded each MVC, with 15 s preceding each sub-maximal contraction. Upon completing the sets of contractions for VA_TMS_ estimation, a final MVC with resting femoral stimulation was performed in order to assess neuromuscular fatigue across the assessment of VA_TMS_. The NMA was repeated, after a small delay (10s), upon completing the single-joint fatiguing exercise (*see* below) to quantify the development of neuromuscular fatigue.

#### Fatiguing exercise

The fatiguing exercise has previously been demonstrated to be effective in inducing rapid reductions in VA_TMS_ [11, 19]. The task consisted of a sustained isometric contraction at 50% MVC for 15 s, followed immediately by 5 s of maximal effort (MVC). This sequence was subsequently repeated following 10 s of rest. During the familiarisation session, the sequence of contractions was performed until task failure, defined as the point at which voluntary torque fell below 50% MVC for >2 s [19]. This sequence lasted 165 s ± 38 s [range: 120–240 s] and was replicated during the experimental trials in order to standardise the exercise load between trials.

### Experimental set-up

Isometric contractions of the right knee extensors (KE) were performed on a multi-joint isokinetic dynamometer (CON-TREX MJ, CMV AG, Dubendorf, Switzerland). Participants sat on the dynamometer with hip and knee angles set at approximately 85° and 90°, respectively (0° = full extension). Extraneous movements of the upper body, thigh and head were constrained as described in Dekerle et al. [11]. A shin-pad attached to the lever arm of the dynamometer was secured to the participant’s leg approximately 3–4 cm proximal to the lateral malleolus. The centre of the rotational axis of the dynamometer was aligned to the axis of the knee joint (lateral femoral epicondyle) before the start of each trial. During KE contractions, participants were instructed to place their arms across their chest, griping the contralateral shoulder strap.

### Torque and Electromyography (EMG)

Isometric torque was digitized (4 kHz) and analysed using LabChart v7.0 software (ADInstruments, Oxfordshire, UK). Surface EMG activity was recorded from the right *vastus lateralis* (VL) and *bicep femoris* (BF) with pairs of self-adhesive electrodes (Kendall H59P, Coviden, Massachusettes, USA) placed according to *SENIAM* guidelines [27]. The reference electrode was placed on the ipsilateral patella. The skin-electrode interface was prepared by shaving the area, lightly abrading and cleansing with a 70% (v/v %) isopropyl alcohol wipe to minimize electrical resistance. The site of electrode placement was optimised to maximise evoked EMG responses and marked with indelible ink to standardise electrode placement between trials. EMG signals were amplified (gain x1000) (PowerLab 26T; ADInstruments, Oxfordshire, UK), digital band-pass filtered (20–2000 Hz), digitized (4 kHz), recorded and later analysed off-line (LabChart v7.0).

### Stimulation techniques

#### Femoral nerve stimulation

Single percutaneous electrical stimuli (duration: 200 μs) were delivered to the right femoral nerve via a pair of square (5 x 5 cm) self-adhesive neuro-stimulation electrodes (Valutrode CF5050; Axelgaard Manufacting Co., Ltd., California, USA), attached to a high-voltage (maximal voltage: 400 V) constant-current stimulator (Model DS7AH, Digitimer Ltd., Hertfordshire, UK). The cathode was placed high in the femoral triangle with the anode positioned midway between the ipsilateral greater trochanter and iliac crest [28]. Precise location of cathode placement was determined through systematic adjustments of the electrode until the greatest twitch torque (Q_pot_) and VL muscle compound action potential (M-wave) amplitude was elicited for a particular sub-maximal current (~70–90 mA) (Johnson *et al*., 2015). This position was recorded and marked with indelible ink for replication between each trial. Optimal stimulation intensity was defined as the intensity at which a plateau in both Q_tw_ and VL M-wave was exhibited. Optimal stimulation intensity was determined through progressive increments in stimulator current (+20 mA) from 10 mA, with two stimuli delivered at each intensity. Stimulation intensity was increased by a further 30% in order to ensure full spatial recruitment of KE motor units. This process was repeated before each trial, with no difference observed between sessions (147 ± 41 mA; 132 ± 40 mA; 146 ± 38 mA; 151 ± 27 mA; F_3,27_: 1.15, *P* = 0.348).

#### TMS

Single, monophasic magnetic stimuli (duration: 1 ms) were manually delivered over the contralateral (left) primary motor cortex, powered by a magnetic stimulator (Magstim^200^, The Magstim Company Ltd., Whitland, UK), using a concave (110 mm) double-cone coil. Orientation of the coil was positioned to induce a posterior-anterior intracranial current flow within the cortex. Optimal coil position (1–2 cm left of vertex) was defined as the site at which the largest motor evoked potential (MEP) was evoked in the VL during a weak contraction (20% MVC) of the KE at 70% maximal stimulator output, with minimal concurrent activation of the antagonist BF, based on the incidental MEP evoked when stimulating the knee-extensors. This site was marked directly onto the scalp with indelible ink. Stimulator intensity during the assessment of VA_TMS_ was selected based on the largest superimposed twitch (SIT) evoked during a brief (~6 s) contraction at 50% MVC [9]. Stimulator output intensity was increased step-wise in 5% increments from 50% of maximal stimulator output until a plateau was reached. Two stimuli were delivered at each intensity during a single contraction, then SIT amplitude was averaged. Each contraction was separated by 15 s rest. The determination of stimulator intensity was conducted prior to each trial, with no difference in mean stimulator output observed throughout the experimental period (66 ± 8%; 65 ± 8%; 65 ± 4%; 65 ± 8%; F_3,27_ = 0.38, *P* = 0.77). The stimulator output activated a large proportion of the KE motoneurone pool in both trials, as evidenced by the comparable MEP/M_max_ ratio during KE MVCs (pre exercise: 54 ± 19%; 45 ± 10%; 44 ± 15%; 42 ± 12%; F_1.59,14_ = 27; P = 0.06). Moreover, this intensity simultaneously evoked small absolute MEP responses in the antagonist BF with a small trial effect (pre-exercise: 1.06 ± 0.91 mV; 0.94 ± 0.76; 1.15 ± 0.76 mV; 0.86 ± 0.71 mV; F_3,27_ = 3.87, P = 0.02); however post hoc tests revealed no significant difference between any trials (*P* ≥ 0.195).

### Data analysis

For all voluntary contractions conducted during the NMA protocols, torque was recorded as the greatest 500 ms average prior to stimulation. Mechanical (i.e. SIT, Q_pot_) and EMG responses (i.e. MEP and M-wave) were analysed for peak-to-peak amplitude following each stimulation. Root-mean-square EMG was quantified as the 500 ms period prior to each stimulation.

VL MEPs were normalised to the electrically evoked EMG response during the maximal contraction (M_max_) preceding the NMA protocol. It has previously been reported that M_max_ is unaffected by increases in voluntary force from 40% to 100% MVC [29], removing the necessity for M_max_ at each voluntary torque level. Absolute antagonist MEP amplitude was assessed at each torque level. The antagonist responses could not be normalised to an appropriate M_max_, due to difficulties in obtaining an M_max_ in the BF [10]. Estimated resting twitch (ERT) was determined as the *y*-intercept of the linear SIT-voluntary torque relationship using least-squares linear regressions (Fig 2). Adjusted *r*^2^ and standard error (SE) associated with slope and *y-*intercept estimates were calculated to examine the goodness of fit of the data to the models. 95% CI (95%CI-ERT) was used as a measure of ERT accuracy. VA_TMS_ was then estimated using Eq 1.

### Statistical analysis

Data are reported as mean ± SD for parametric sets unless stated otherwise. Normal Gaussian distribution set was verified for each data using the Shapiro-Wilk test. Three-way ANOVAs with repeated measures were performed to assess effects for session (session 1 *vs* 2), exercise (pre- *vs* post-exercise), and, depending on the research question, either model used (3 *vs* 5 *vs* 10 data points) or NMA protocol (pre- and post-NMA). Sphericity was assessed using Mauchly’s test. When the assumption of sphericity was violated, the significance of F-ratios was adjusted according to the Greenhouse–Geisser procedure. Effect sizes are presented as partial eta squared (η_*p*_^2^) for main and interaction effects.

Absolute reliability was assessed through calculation of Typical Error of Measurement (Eq 2) sometimes named ‘Standard Error of Measurement’ [30]. Systematic biases and random errors were assessed from Bland and Altman plots [31]. Heteroscedasticity was examined by plotting absolute differences against individual means with subsequent calculation of Pearson correlation coefficient following prior check for normal Gaussian distributions (heteroscedasticity correlation coefficient, HCC). HCC was used to assess the significance of the relationships. If heteroscedasticity was detected or the differences not normally distributed, the data were logarithmically transformed. In a second step, heteroscedasticity and normal Gaussian distribution were tested from the log-transformed data. The 95% absolute or ratio limits of agreement were calculated accordingly. Relative reliability was quantified through calculation of Intraclass Correlation Coefficient (two-way random effect; A,1; [32]). Due to the ceiling effect associated with the measure of cortical VA, ICC was not calculated for this variable [33].

$$TEM=\frac{SD\;of\;individual\;differences}{\sqrt{2}}$$ (2)

The smallest detectable change or the minimum chance for a change likely to be ‘real’ (P<0.05) for one individual was also calculated for each key variable (Eq 3; [34]). To be noted, SDC is the same as the 95% limit of agreement from the Bland and Altman plot. Sample’s SDC values were derived from SDC_ind_ [34]. Responsiveness of the key measures of neuromuscular fatigue was ascertained for each participant and for the sample of participants when an individual pre- to post-intervention difference (Δ change) and the mean change in the individual differences (Δ change in the mean) were greater than SDC_ind_ and SDC_sample,_ respectively.

$${SDC}_{ind}=1.96\;x\;\sqrt{2}\;x\;SEM$$ (3)

All statistical procedures were performed using SPSS (version 22, Chicago, USA) with the null hypothesis rejected at an alpha level of 0.05.

## Results

### Linear relationship between SIT and voluntary torque and accuracy in estimates

Absolute torques and associated SITs for the 2x5C protocol are shown in S1 Table (*see* Table 1 in Dekerle et al., [11] for the 3x3C protocol). There were no significant differences between session 1 and 2 for both voluntary torque (*F*_*1*,*9*_ = 0.233, *P* = 0.64, η_*p*_^2^ = 0.025) but SIT values were significantly greater for the second session (*F*_*1*,*9*_ = 7.56, *P* = 0.02, η_*p*_^2^ = 0.457). No interaction was significant when looking for any of the session-combined effects (*P*>0.05).

All 10-point relationships from the 2x5C protocol were significantly linear (*P* < 0.05). Linearity was however not confirmed (*P* > 0.05) in 22.5% of the 5-point relationships: 7 of 40 relationships (17.5%) for the first set of 1x5C (pre- exercise: 2/20; post-exercise: 5/20); 11 of 40 relationships (27.5%) for the second set of 1x5C (pre- exercise: 6/20; post-exercise: 5/20). Six of these non-linear relationships belonged to the same participant with visual inspection identifying no outlier for five of these relationships so the participant was excluded for some analyses (*n* = 9). Visual inspection of the remaining 12 non-significant relationships (pre-exercise: 1/20 for both set 1 and 2; post-exercise: 4/20 and 6/20 for set 1 and 2) allowed for the identification of one outlier in the linear relationship. Once removed, the linearity of the resultant 4-point regressions was significant (*P*<0.05). As a consequence, all first sets of 5 contractions for the 2x5 protocol could be modelled.

A more in-depth comparison with 3x3C (S1 Table) shows no difference in SIT values across the 50–100% levels of contractions between the two protocols. Reports of linearity outcomes for the 1x3C and 3x3C protocols are detailed in Dekerle et al. [11]. In summary, the linearity of 1x3C relationships was only statistically significant (*P* < 0.05) for 16 of 120 relationships (13%). In all but one instance (post-exercise), the nine-point linear regression was significant (*P* < 0.05). Removal of one identified outlier in the non-linear data set (a SIT at 50%MVC; >1.96 SD from casewise diagnostic) led to a significant relationship (*r*^2^ = 0.61; *P* = 0.02), with an 8-point regression used for ERT determination as a consequence.

Comparison between the various models (Table 2) revealed no significant difference in the adjusted *r*^2^ (*F*_*6*,*42*_ = 2.84, *P* = 0.07, η_*p*_^2^ = 0.29) but differences in the accuracy of ERT (SE-ERT: *F*_*6*,*42*_ = 6.9, *P* < 0.01, η_*p*_^2^ = 0.49; 95% CI-ERT: *F*_*6*,*42*_ = 34.0, *P* < 0.01, η_*p*_^2^ = 0.83). 95% CI-ERT was enhanced significantly when adding two SIT values in the modelling (1x5C *vs* 1x3C: P = 0.001), with no statistical change when increasing the number of SIT values (1x5C *vs* 2x5C: P = 0.338; 1x5C *vs* 3x3C: P = 0.810; 2x5C *vs* 3x3C: P = 1).

**Table 2:** Adjusted r^2^ and 95% CI associated with ERT for the linear regressions between SIT and voluntary torque Session 1 and 2 averaged).

	Pre-exercise	Post-exercise		
	**Adjusted *r***^**2**^			
2x5C	0.83 ± 0.12	0.76 ± 0.14		
3x3C	0.88 ± 0.06	0.74 ± 0.19		
1x5C –set 1	0.87 ± 0.08	0.89 ± 0.07		
1x5C –set 2	0.92 ± 0.05	0.92 ± 0.08		
1x3C –set 1	0.85 ± 0.11	0.68 ± 0.40		
1x3C –set 2	0.86 ± 0.13	0.69 ± 0.36		
1x3C –set 3	0.87 ± 0.10	0.80 ± 0.22		
	**± 95% CI–ERT (N.m (%ERT))**			
2x5C	± 8.6 (25%)	± 5.3 (24%)	*	
3x3C	± 8.2 (23%)	± 5.5 (29%)	*	
1x5C –set 1	± 16.5 (46%)	± 8.7 (40%)	*	
1x5C –set 2	± 14.0 (41%)	± 7.7 (39%)	*	
1x3C –set 1	± 94.8 (257%)	± 45.6 (264%)	*	#
1x3C –set 2	± 88.0 (255%)	± 56.4 (283%)	*	#
1x3C –set 3	± 81.6 (235%)	± 34.7 (169%)	*	#

* Significantly different from pre- to post-exercise (P<0.05)

# Significantly different from 2x5C and 1x5C models (P<0.05)

### Internal validity of the 2x5C NMA protocols

Results for the study of internal validity of the NMA protocols are presented in Table 3. At baseline, the NMA resulted in a significant decline in MVC (F_1,9_ = 11.625, P = 0.008, η_*p*_^2^ = 0.564) and Q_pot_ (F_1,9_ = 14.616, P = 0.004, η_*p*_^2^ = 0.619) following 2x5C protocol. Both variables significantly recovered during the NMA performed post-exercise (MVC: F_1,9_ = 23.472, *P* = 0.001, η_*p*_^2^ = 0.723; Q_pot_: F_1,9_ = 3.227, P = 0.001, η_*p*_^2^ = 0.830). In total, the number of contractions performed during the assessment of VA_TMS_ was 14 and the NMA protocols lasted 287 s protocol. A more in-depth comparison with 3x3C (S1 Table) shows changes of similar magnitudes between the two protocols.

**Table 3:** Means, standard deviations, and percent changes in key measures of the neuromuscular function before, during, and after the 5C protocol.

	Pre-exercise			Post-exercise			
	**Pre-NMA**	**Post-NMA**	**%Change**	**Pre-NMA**	**Post-NMA**	**% Change**	
MVC	243 ± 46 N.m*	228 ± 44 N.m^*#*^	-6.3 ± 4.3	174 ± 29 N.m*	195 ± 40 N.m^*#*^	+11.3 ± 8.6	
Q_pot_	59 ± 7 N.m*	56 ± 9 N.m^*#*^	-6.2 ± 6.6	34 ± 7 N.m*	42 ± 7 N.m^*#*^	+23.1 ± 12.5	
	**Set 1**	**Set 2**	**%Change**	**Set 1**	**Set 2**	**% Change**	
Q_pot_	59 ± 8 N.m*	58 ± 7 N.m^*#*^	+1.1 ± 3.3	37 ± 7 N.m*^,^^*#*^	40 ± 6 N.m^*#*^	+8.5 ± 12.2	
Slope of the linear relationship	-0.16 ± 0.05	-0.15 ± 0.05	-3.8 ± 16.4	-0.12 ± 0.06	-0.11 ± 0.06	-18.7 ± 25.6	^$^
*y*-intercept of the linear relationship	36.1 ± 8.2 N.m	33.9 ± 7.9 N.m	-4.5 ± 12.9	21.6 ± 8.6 N.m	19.7 ± 9.7 N.m	-14.8 ± 19.4	^$^
VA_TMS_	94 ± 4%	95 ± 4%	-0.8 ± 6.1	86 ± 8%	83 ± 11%	-3.5 ± 13.9	

* Significantly different from post-NMA (P<0.05)

# Significantly different from pre-NMA (P<0.05)

$ Significantly different between 1^st^ vs 2^nd^ set of 1x5C of the 2x5C protocol (P<0.05)

To investigate changes in the neuromuscular function after the first set of 1x5C, a Q_pot_ was evoked after the 100% MVC of each 1x5C (*see* Fig 2). The two Q_pot_ were not significantly different (*F*_*1*,*7*_ = 0.23, *P* = 0.65, η_*p*_^2^ = 0.03). Both slope (*F*_*1*,*16*_ = 16.46, *P* < 0.01, η_*p*_^2^ = 0.51) and *y*-intercept (*F*_*1*,*16*_ = 15.0, *P* < 0.01, η_*p*_^2^ = 0.48) were significantly different between the two sets of 1x5C (*n* = 8). Post-hoc analysis did not reveal differences pre-exercise (Table 3; *P* > 0.05) but values were found significantly lower for set 2 post-exercise (*y*-intercept: -9%; *P* < 0.01; slope: -15%; *P* < 0.01; Table 3). Interestingly, VA_TMS_ was not significantly different between set 1 (94 ± 4% and 86 ± 8% pre- and post-exercise) and set 2 (95 ± 5% and 83 ± 11% pre- and post-exercise) (*F*_*1*,*7*_ = 1.578, *P* = 0.25, η_*p*_^2^ = 0.18).

### Reliability and interchangeability

**Fig 3:**
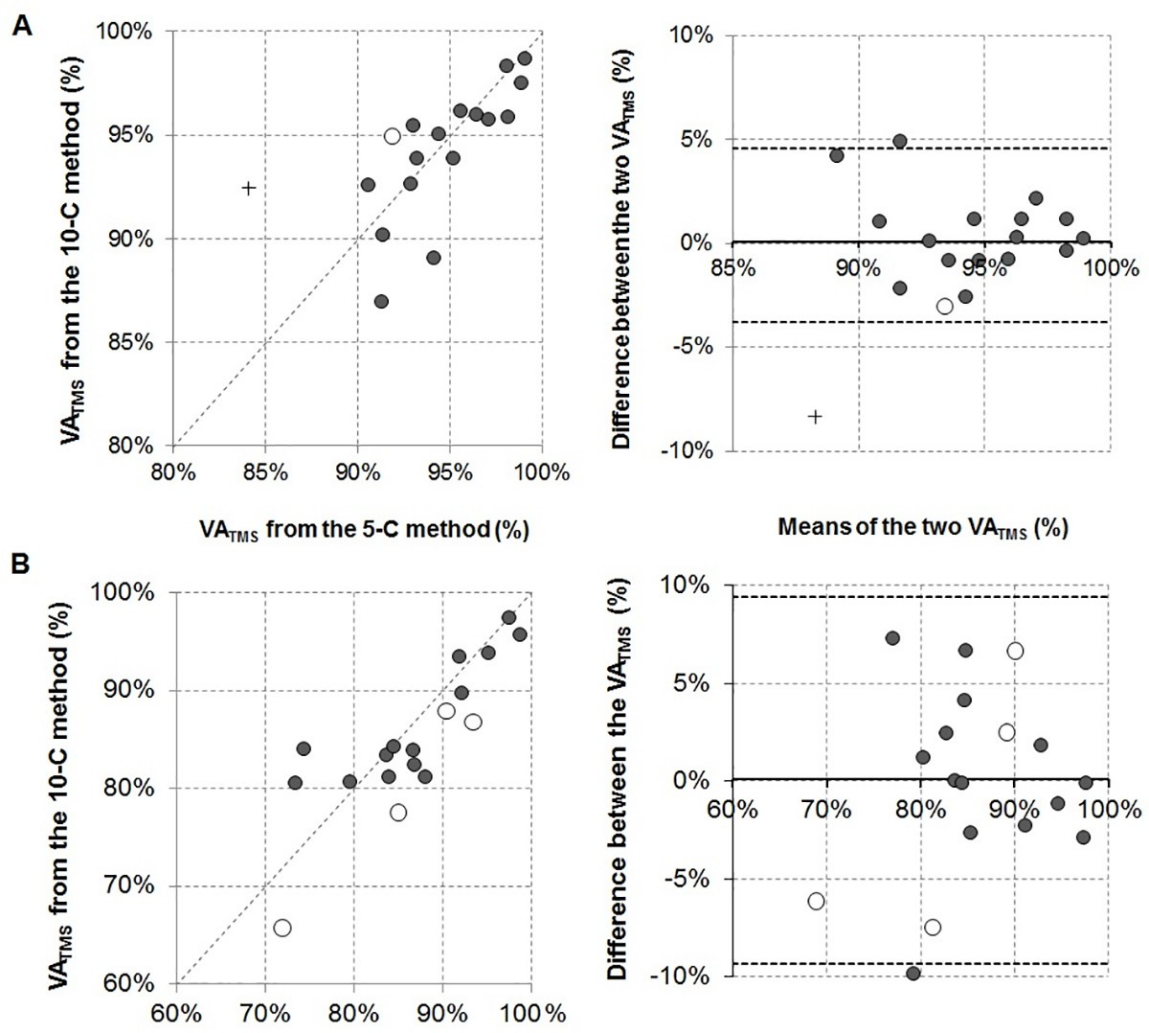
Scatter plots and Bland and Altman plots for the comparison of VA_TMS_ using the 5-C method (Panel A, pre-exercise; Panel B, post-exercise). Panel A and B: White circles illustrate VA_TMS_ values for which ERT had to be extrapolated from a 4-point relationship (for 1x5C only). Panel A: The cross shows an outlier that has to be removed to allow for the calculation of bias ± 95% LA.

Reliability measures for 1x5C and 2x5C are presented in Table 4. There was no significant difference between the two 2x5C sessions for ERT, SIT_100%_ and VA_TMS_ whether they were calculated using the 1x5C or 2x5C linear model (P>0.05). Only ERT was significantly different between the 1x5C and 2x5C models (*F*_*1*,*8*_ = 11.6, *P* = 0.009, η_*p*_^2^ = 0.59). However, this slight difference was not large enough to fall outside the SDC_sample_. For the baseline measures, VA_TMS_ of 1x5C was significantly correlated to VA_TMS_ of 2x5C (ICC_2,1_ = 0.65; *P* = 0.002). Bias ± 95% LA could not be calculated since neither differences between the two sets of VA_TMS_, nor their log transformation, were normally distributed (*P*>0.05). However, removal of an outlier (*see* Fig 3, Panel A) led to bias ± 95% LA of 0.4 ± 4.2%. Post-exercise, VA_TMS_ of 1x5C was significantly correlated to those of 2x5C (ICC_2,1_ = 0.82; *P*<0.001) with bias ± 95% levels of agreement of 0.1 ± 9.4%.

**Table 4:** Between-session Mean ± SD and reliability data for the determination of VA_TMS_ using the first 5 (n = 8) and all 10 contractions for the estimation of ERT (n = 10).

		Session 1 Mean ± SD	Session 2 Mean ± SD	TEM (% of mean)	Bias ± 95% LA	SDC_ind_ (% of mean)	SDC_sample_ (% of mean)	ICC_2,1_ (95%CI)		
**Pre-exercise**										
**ERT**	5	36.0 ± 8.4	35.7 ± 8.8	3.8 (10.5%)	-0.4 ± 10.4 ^(H0)^	10.4 (29.1%)	3.5 (9.7%)	.83* (.39-.96)		#
In N.m	10	34.0 ± 7.0	35.9 ± 8.2	3.8 (10.9%)	1.9 ± 10.6 ^(H0)^	10.6 (30.2%)	3.3 (9.5%)	.85* (.45-.96)		
**SIT100%**	5	2.0 ± 1.0	1.9 ± 1.3	0.7 (37.2%)	-0.1 ± 2.0 ^(H0)^	2.0 (103%)	0.4 (34.4%)	.60* (-.19-.94)		
In N.m	10	1.8 ± 0.8	2.2 ± 1.4	0.8 (40.7%)	0.1 ± 1.0	2.2 (113%)	0.7 (35.7%)	.63^n.s.^ (-.34-.91)		
**VA**_**TMS**_	5^.^	94.0 ± 2.8	94.2 ± 4.6	3.0 (3.2%)	0.2 ± 8.3 ^(H0)^	8.3 (8.8%)	2.8 (2.9%)	n.a.		
In %	10	94.7 ± 2.3	93.8 ± 3.7	2.4 (2.6%)	0.01 ± 0.07	6.7 (7.1%)	2.1 (2.3%)	n.a.		
**Post-exercise**										
**ERT**	5	19.6 ± 6.3	24.3 ± 10.5	4.8 (21.8%)	4.7 ± 13.2 ^(H0)^	13.2 (60.3%)	4.4 (20.1%)	.77* (.08-.95)	$	#
In N.m	10	17.9 ± 7.3	22.5 ± 9.6	4.2 (20.9%)	4.6 ± 11.7 ^(H0)^	11.7 (57.8%)	3.7 (18.3%)	.81* (.18-.95)		
**SIT100%**	5	2.6 ± 2.0	2.8 ± 1.3	1.4 (52.3%)	0.3 ± 1.5	3.9 (145%)	1.3 (48.3%)	.45* (-.54-.91)	$	
In N.m	10	2.5 ± 1.4	3.0 ± 1.2	0.9 (34.3%)	0.5 ± 2.6 ^(H0)^	2.6 (95.1%)	0.8 (30.1%)	.63 ^n.s.^ (-.29-.90)		
VA_TMS_	5	86.0 ± 9.5	86.9 ± 6.6	8.4 (9.7%)	0.8 ± 23.2 ^(H0)^	23.2 (26.6%)	7.7 (9.0%)	n.a.	$	
In %	10	84.5 ± 9.2	86.9 ± 6.6	6.8 (8.0%)	0.8 ± 18.8 ^(H0)^	18.8 (22.1%)	5.9 (7.0%)	n.a.		

^(H0)^ Homoscledasticity verified (*P*<0.05)

^n.s.^ No statistical significance (*P*>0.05)

*Statistical significance (*P*>0.05)

*n*.*a*. for non-applicable (ceiling effect for ICC); $ Statistical significance from pre- to post-exercise (*P*<0.05); # Statistical significance from the 10-point modelling (*P*<0.05); SDC_ind_ smallest detectable change for an individual participant; SDC_sample_ smallest detectable change for the sample; ICC intraclass correlation coefficient.

For each level of contraction, RMS.M_max_^-1^ did not differ between sessions (P>0.05) but decreased significantly post-exercise (P<0.05). This was accompanied with no difference in M_max_ between the two sessions (F_(1,9)_ = 0.163, P = 0.695, np^2^ = 0.018) but an increase pre- to post-exercise (F_(1,9)_ = 6.346, P = 0.033, np^2^ = 0.414).

### Sensitivity–Effect of the fatiguing exercise

All markers of neuromuscular fatigue were reduced following the fatiguing isometric exercise: -28% for MVC (F_(1,9)_ = 71.13, P<0.001, η_*p*_^2^ = 0.89), -41% for Q_pot_ (F_(1,9)_ = 93.1, P<0.001, η_*p*_^2^ = 0.92), -38% (1x5C) or -43% (2x5C) for ERT (1x5C: F_1,9_ = 76.04, *P* < 0.001, η_*p*_^2^ = 0.89; 2x5C: F_1,9_ = 1.7.86, *P* < 0.001, η_*p*_^2^ = 0.92; Tables 3 and 4), and -8% (1x5C) or -10% (2x5C) for VA_TMS_ (1x5C: F_1,9_ = 32.97, *P* < 0.001, η_*p*_^2^ = 0.79; 2x5C: F_1,9_ = 40.83, *P* < 0.001, η_*p*_^2^ = 0.82). Individual changes in VA_TMS_ from pre-exercise were deemed detectable in 35% and 75% of the individuals for the 1x5C and 2x5C method, respectively. The greater sensitivity of the 10-point method was not so evident when considering individual changes from post-exercise values (‘true’ change detected in 5% of the individuals for both methods). Changes in the sample’s means for VA_TMS_ from pre- to post-exercise were deemed detectable in all cases (session 1 and session 2), for both methods.

## Discussion

The main aim of the present study was to examine the accuracy, validity, reliability and sensitivity of two novel NMAs (1x5C and 2x5C) designed to improve the measurement of neuromuscular fatigue following high intensity exercise. The present data shows the significant improvement in ERT accuracy when the number of points included in the linear modelling of SIT vs voluntary contraction is increased. Neuromuscular fatigue (MVC and Qpot) is evident following 2x5C (and 3x3C; more details in S1 Text, S1 Table, S2 Text, and Dekerle et al., [11]) but not 1x5C. Conversely, neuromuscular recovery takes place during both 1x5C and 2x5C (and 3x3C; more details in S1 Text, S1 Table, S2 Text, and Dekerle et al., [11]). However, these NMA-induced effects do not affect VATMS: Both 1x5C and 2x5C provide reliable measures of all neuromuscular outcomes, and VATMS more importantly. Both protocols detected ‘true’ change in VATMS induced by the fatiguing exercise for the sample of participants tested. ‘True’ Individual changes however could not be identified with such certainty.

### Goodness of fit of the linear modelling of the SIT-voluntary torque relationship

As predicted, ERT accuracy improves greatly when as few as two extra SITs are input into the linear regression of the SIT-voluntary contraction relationship: CI-ERT improved by six fold when five as opposed to three data points are included in the linear regressions, with no further statistical improvement when doubling the number of SITs (1x5C *vs* 2x5C). This approach for more data points entered in the model had already been considered and/or adopted in both early and more recent studies [5, 19] but had gained little traction. This may be because accuracy in ERT was not examined closely. Researchers have used a 1x3C protocol [6] or a three-point relationship to estimate RT [12], likely for the purpose of measuring VA_TMS_ as soon as possible following exercise. CI-ERT in these studies would have been within the range of ~250% of ERT (Table 2). Authors have conventionally adopted an arbitrary *r* threshold of < 0.9 as acceptable linearity [35]. Conversely in the present findings, adjusted *r*^2^, an index of variance explained by the best fit, and not an estimate of ERT accuracy [36], was not affected by the number of data points entered in the model (Table 2). This demonstrates the limitations of *r*^*2*^ as the sole criterion to assess the goodness of fit of a linear relationship [36], and the need for a more valid measure of accuracy for the main estimates (i.e. 95% CI–ERT), especially when a high accuracy is sought in the subsequent determination of VA_TMS_. With poor 95%CI-ERT when using a three-point linear model to estimate RT, the present findings are in line with previous report of a lack of significant linearity of this model [11] and strengthen the claim that the use of a three-point linear model should be avoided for ERT determination. SIT measurements over a range of at least five levels of contractions should therefore be recommended.

### Internal validity, reliability, and sensitivity of the NMA protocols at baseline

Neuromuscular function was affected by the 2x5C NMA protocol, with a loss of force-generating capacity following NMA at baseline (-6% in MVC). This could be explained by disturbances distal to the neuromuscular junction (-6% in Q_pot_) caused by either fatigue-inducing mechanisms or a loss of potentiation [37, 38]. These changes were greater than the SDC_sample_ [11] and were similar to those observed following the traditional 3x3C NMA protocol [11] despite the 2x5C protocol requiring one more contraction and taking place over a longer period of time (+ 8 s). However, these small peripheral impairments were not observed after one 1x5C only (Table 3) suggesting the 1x5C NMA protocol holds robust internal validity for baseline measurement. Despite these peripheral disturbances during the course of the NMA, slope and *y*-intercept of the SIT-voluntary torque relationship were not altered by the performance of a second set of 1x5C (Table 3) so that VA_TMS_ was not different between the two sets of 1x5C, and with 2x5C. The measure was found extremely stable from one session to the other (TEM of 3.0% for 1x5C and 2.4% for 2x5C) as previously shown for the 3x3C protocol: TEM of 1.7% in [4] and 2.3% [11]. Both protocols (1x5C and 2x5C) therefore allow for the mean change in VA_TMS_ following the fatiguing exercise (loss of 8–9%) to be detected (>SDC_sample_). This was the case for both sessions while the 3x3C protocol only depicted a detectable change in one of the two sessions [11]. Therefore, either the 1x5C or the 2x5C protocol may be chosen as an alternative to the 3x3C protocol to assess changes in VA_TMS_ from a resting state with the present findings evidencing possible interchangeability between the two protocols (bias ± 95% LA of 0.4 ± 4.2%).

### Internal validity, reliability, and sensitivity of the NMA protocols following fatiguing exercise

In agreement with previous findings [11, 24], all neuromuscular outcomes were less reliable when assessed after the performance of a fatiguing exercise (Table 4). TEM for VA_TMS_ increased (x 2.8) to 8.4% and 6.8% for the 1x5C and 2x5C protocol, respectively. This remained however slightly better than TEM for the 3x3C protocol (9.8%), which was also found to be less sensitive to both individual and sample’s changes in VA_TMS_ following the fatiguing exercise (Dekerle et al. [11]).

These findings regarding VA_TMS_ were obtained despite a recovery of the neuromuscular function during the NMA protocol (+11% MVC from pre- to post-NMA), with a concomitant bi-phasic recovery at the periphery: Peripheral fatigue initially recovered very quickly in the first part of the NMA protocol (+8% Q_pot_ in 30 seconds (from 70^th^ to 100^th^ second post-exercise); Table 3 and Fig 1), before a slower recovery phase (+7% and +4% Q_pot_ over the 110 seconds of 1^st^ and then 2^nd^ set of 1x5C). Similar recovery patterns have been reported in the literature, with a plateau in the recovery of twitches evoked via paired (10 and 100 Hz) and tetanic stimuli from 4 minutes following high-intensity isokinetic knee extension / flexion exercise [18]. Partial recovery of peripheral fatigue has also been evidenced within the first 6 minutes following a 2-minute MVC in the knee extensors [6]. One may at this point, discuss the statistical significance in the difference in ERT (an estimate of contractile function) between the two sets of 1x5C. But the magnitude i such (<SDC_sample_) that it should not be seen as a ‘true detectable change’. Taken together, our findings confirm the rapid recovery in peripheral fatigue during a NMA conducted after a fatiguing exercise [11]. This represents a confounding factor that ought to be considered when interpreting NMA outcomes. This recovery of neuromuscular fatigue (+11% in post-NMA MVC) through putative mechanisms occurring distally to the neuromuscular junction (+23% in post-NMA Q_pot_) was of similar magnitude between the 3x3C and 2x5C protocols (more details in S1 Text, S1 Table and S2 Text). However, these changes were smaller after 1x5C, indicating that the 1x5C presented is a more valid protocol for the study of exercise-induced neuromuscular fatigue. The Bland and Altman Plot (Fig 3, Panel B) illustrates the small mean difference (bias of 0.1%) between VA_TMS_ calculated using 1x5C *vs*. 2x5C, but with larger inter-individual variability in these differences (95% LA of 9.4%). These may be attributed to *(a)* the variability in SIT_100%_ (*see* Table 4 and Dekerle et al. [11], *(b)* individualities in the patterns of recovery taking place within the 2x5C protocol, and *(c)* the removal of one SIT outlier for four of the 1x5C models for the extrapolation of ERT from the SIT–voluntary torque relationship (white circles in Fig 3). Interesting, when these four VA_TMS_ values were removed (*n* = 14), bias ± 95% LA (0.4 ± 8.5%) remained similar between the two sets of VA_TMS_.

### Limitations and future directions

In the present study, the SIT—voluntary torque relationship was assumed to be linear and modelled accordingly. Of interest is the almost systematic non linearity for one of our participants (6 of 8 relationships) with no apparent explanation for this observation. Both pre- and post-exercise, there was a tendency in some cases for the SIT–voluntary torque relationship to display a concave curvilinear shape (*see* S1 Fig). Curvilinear shapes have been reported in the literature for SITs evoked via percutaneous electrical nerve stimulation [38–40]. The shape of the SIT–voluntary torque relationship remains controversial with various methodological and physiological factors that have been shown or suggested to distort its shape [20, 41]. Some of these factors may apply for the TMS-evoked SITs. Todd et al. [1] reported, both in the fresh and fatigued elbow flexors, linearity in the relationship across the range of 50 to 100% of MVC, but curvilinearity for SITs evoked via electrical motor nerve stimulation. This is an accepted core assumption for the measure of VA_TMS_ which may warrant further investigation.

The performance of three MVCs in the first part of the NMA protocol was chosen to facilitate potentiation of the evoked potentiated twitch as suggested by (Kufel et al. [26]) but may have induced an early development of neuromuscular fatigue. The present VA_TMS_ values at baseline (94% and 95% for set 1 and 2 of 1x5C) are indeed consistent with other measures performed after a succession of three MVCs [8, 24]. They seem slightly greater than following six successive MVCs (92–93% [23]). But they may appear slightly lower than when only one MVC is performed before the subsequent set of sub-maximal contractions (around 98%; [6, 19]).

Delays in NMA initiation (10s) and rests between contractions (15s) may have resulted in substantial recovery of VA_TMS_ (Mira et al., 2017), such that VA_TMS_ is underestimated in the present study. A protocol that combines rapid measure of VA_TMS_ as proposed by Mira et al (2017) with robustness of 1x5C or 2x5C protocols, could be investigated further.

Most but not all 1x5C relationships were significant (1/5 ratio). However, visual inspections allowed for outliers to be identified and removed with the 1x5C method, and the linearity of all but one of the 1x4C relationships then became significant. This approach reduces ERT accuracy but remains an option the 1x3C method does not offer. All SIT–voluntary contraction relationships were significant when 9 points (3x3C; Dekerle et al. [11]) or 10 points (2x5C) were entered into the model. A 1x6C protocol could warrant further investigation and become a good compromise between accuracy and reliability *vs* internal validity.

## Conclusion

The accuracy in ERT improves greatly when increasing the number of SITs measures input into the linear regression of the SIT-voluntary contraction relationship: CI-ERT is six fold lower when five as opposed to three data points are included in the linear regressions, with no further statistical improvement when doubling the number of SITs (1x5C *vs* 2x5C). Besides better accuracy, both 1x5C and 2x5C also provide more reliable measures of VA_TMS_ at baseline and post-exercise, with an enhanced sensitivity to the fatiguing exercise when compared to the more traditional 3x3C protocol. The 2x5C protocol may be marginally more sensitive to individual changes. However, the 1x5C protocol holds stronger internal validity both at baseline and following a fatiguing exercise and may therefore be chosen for capturing exercise-induced fatigue.

## Supporting information

S1 Data set Data.xlsx: Data from the two experimental visits, pre and post exercise. (XLSX)

S1 Text Internal validity of 5x5C *vs* 3x3C protocols. (DOCX)

S2 Text Statistical analysis of torque measures recorded during voluntary contractions (100%, 87.5%, 75%, 62.5%, and 50% of MVC) and TMS-evoked superimposed twitches during the 5-C NMA performed before and after a fatiguing task. (DOCX)

S1 Table Means ± Standard Deviations of torque recorded during voluntary contractions (100%, 87.5%, 75%, 62.5%, and 50% of MVC) and TMS-evoked superimposed twitches during the 5-C NMA performed before and after a fatiguing task. (DOCX)

S1 Fig Example of a curvilinear relationship for one participant. (DOCX)

## Abbreviations

ERT: Estimated resting twitch
ICC: Intraclass correlation
KE: Knee extensors
MEP: Motor evoked potential
MVC: Mximal voluntary contractions
NMA: Neuromuscular assessment
POT: Potentiated twitch force
SDC: Smallest detectable change
SIT: Superimposed twitch
SIT_100%_: Superimposed twitch during a maximal voluntary contraction
TMS: Transcranial magnetic stimulation
VA: Voluntary activation
VA_TMS_: Voluntary activation using transcranial magnetic stimulation
VC: Voluntary contraction
