# Supplementary material for: Improving the measurement of TMS-assessed voluntary activation in the knee extensors
Source: PLoS One. 2019 Jun 6;14(6):e0216981. doi: 10.1371/journal.pone.0216981 (PMC6553714; doi:10.1371/journal.pone.0216981)
Supplement: S1 Text — (DOCX) [file pone.0216981.s002.docx]

**S1 Text: Internal validity of 5x5C *vs* 3x3C protocols**

With no difference between sets of contractions for both protocols, an average of two (5C) and three (3C) values was calculated for each contraction level: SIT values recorded at 100%, 75% and 50% of MVC were not significantly different between the two protocols (*F_1,9_*= 0.427, *P* = 0.53, η*_p_*^2^= 0.045) despite the small time difference between protocol (+5 s) in the measures at 75% and 50% of MVC during the 2x5C protocol. There was also no significant interaction for any of the protocol-based interaction effect (*P* > 0.05).

At baseline, the NMA resulted in a significant decline in MVC (F_1,9_ = 11.625, P = 0.008, η_p_^2^ = 0.564) and Q_pot_ (F_1,9_ = 14.616, P = 0.004, η_p_^2^ = 0.619) following both 2x5C and 3x3C protocols. Post-exercise, MVC and Q_pot_ significantly recovered from pre- to post-NMA (MVC: F_1,9_ = 23.472, P = 0.001, η_p_^2^ = 0.723; Q_pot_: F_1,9_ = 3.227, P = 0.001, η_p_^2^ = 0.830). These changes were not significantly different between 3x3C and 2x5C whether measured pre-exercise (MVC: -13 vs. -15 N.m, F_1,9_ = 0.714, P = 0.412, η_p_^2^ = 0.076; Q_pot_: -3 vs. -4 N.m, F_1,9_ = 0.882, P = 0.500, η_p_^2^ = 0.052) or post-exercise (MVC: 5 vs. 21 N.m, F_1,9_ = 1.659, P = 0.230, η_p_^2^ = 0.156; Q_pot_: 7 vs. 7 N.m, F1,7 = 1.127, P = 0.139, η_p_^2^ = 0.324). In total, the number of contractions performed during the assessment of VATMS was 14 and 13, and the NMA protocols lasted 287 s and 279 s, for the 2x5C and 3x3C protocol, respectively.

Of interest, while the two Q_pot_ evoked after each 100%MVCs during 2x5C were not significantly different (*F_1,7_*= 0.23, *P* = 0.65, η*_p_*^2^= 0.03), the three Q_pot_ recorded within 3x3C were not significantly different either (*F_2,18_*= 0.44, *P* < 0.55, η*_p_*^2^= 0.047).
