# Supplementary material for: Improving the measurement of TMS-assessed voluntary activation in the knee extensors
Source: PLoS One. 2019 Jun 6;14(6):e0216981. doi: 10.1371/journal.pone.0216981 (PMC6553714; doi:10.1371/journal.pone.0216981)
Supplement: S2 Text — (DOCX) [file pone.0216981.s003.docx]

**S2 Text: Statistical analysis of torque measures recorded during voluntary contractions (100%, 87.5%, 75%, 62.5%, and 50% of MVC) and TMS-evoked superimposed twitches during the 5-C NMA performed before and after a fatiguing task.**

There were no significant differences between session 1 and 2 for voluntary torque (*F_1,9_* = 0.233, *P* = 0.64, η*_p_*^2^ = 0.025) and for any interaction effects (*P*>0.05). Voluntary torque was not significantly different between set 1 and 2 (*F_1,9_* = 3.93, *P* = 0.08, η*_p_*^2^ = 0.304) and the insignificance remained when considering exercise *x* set interaction (*F_1,9_* = 1.19, *P* = 0.30, η*_p_*^2^ = 0.117) and contraction *x* set interaction (*F_1,9_* = 1.59, *P* = 0.19, η*_p_*^2^ = 0.151). Values were significantly lower (*F_1.9_* = 66.9, *P* < 0.01, η*_p_*^2^ = 0.882) and decreased less greatly post-exercise (*F_4,36_* = 53.6, *P* < 0.01, η*_p_*^2^ = 0.856). SIT values were significant different between session 1 and 2 (*F_1,9_* = 7.56, *P* = 0.02, η*_p_*^2^ = 0.457) but with no other interaction effect when considering session effect (*P*>0.05).

SIT were not significantly different between set 1 and 2 (*F_1,9_* = 1.343, *P* = 0.28, η*_p_*^2^ = 0.13) with no other interaction effect when considering set effect (*P*>0.05). SIT values were significantly greater post-exercise (*F_1.9_* = 18.60, *P* < 0.01, η*_p_*^2^ = 0.674), decreased significantly as the level of contraction increased (*F_4,36_* = 86.4, *P* < 0.01, η*_p_*^2^ = 0.906) with a lesser decrease post- compared to pre-exercise (*F_4,36_* = 51.2, *P* < 0.01, η*_p_*^2^ = 0.851).
