# Supplementary material for: Improving the measurement of TMS-assessed voluntary activation in the knee extensors
Source: PLoS One. 2019 Jun 6;14(6):e0216981. doi: 10.1371/journal.pone.0216981 (PMC6553714; doi:10.1371/journal.pone.0216981)
Supplement: S1 Table — (DOCX) [file pone.0216981.s004.docx]

S1 Table: Means ± Standard Deviations of torque recorded during voluntary contractions (100%, 87.5%, 75%, 62.5%, and 50% of MVC) and TMS-evoked superimposed twitches during the 5-C NMA performed before and after a fatiguing task.

|  |  | **100%** | **87.5%** | **75%** | **62.5%** | **50%** |  |  |  |
| --- | --- | --- | --- | --- | --- | --- | --- | --- | --- |
| **Visit 1**  **Pre** | Set 1 - Voluntary Contractions (N.m^-1^) | 231 ± 45 | 205 ± 39 | 178 ± 33 | 148 ± 28 | 121 ± 24 |  | ^$^ |  |
|  | Set 2 - Voluntary Contractions (N.m^-1^) | 230 ± 44 | 205 ± 40 | 177 ± 33 | 147 ± 27 | 120 ± 23 |  | ^$^ |  |
|  | Set 1 - SIT (N.m^-1^) | 2.0 ± 0.9 | 3.1 ± 1.6 | 6.0 ± 2.7 | 11.2 ± 4.1 | 19.2 ± 6.0 |  | ^$^ | ^#^ |
|  | Set 2 - SIT (N.m^-1^) | 1.6 ± 1.1 | 3.0 ± 1.6 | 5.9 ± 2.6 | 12.5 ± 4.4 | 16.1 ± 4.0 |  | ^$^ | ^#^ |
| **Visit 1** | Set 1 - Voluntary Contractions (N.m^-1^) | 171 ± 36 | 148 ± 30 | 127 ± 27 | 107 ± 22 | 85 ± 16 | ^*^ | ^$^ |  |
| **Post** | Set 2 - Voluntary Contractions (N.m^-1^) | 171 ± 37 | 148 ± 34 | 129 ± 28 | 107 ± 21 | 86 ± 17 | ^*^ | ^$^ |  |
|  | Set 1 - SIT (N.m^-1^) | 2.4 ± 1.9 | 2.7 ± 1.3 | 5.3 ± 2.2 | 7.0 ± 1.8 | 10.5 ± 4.1 | ^*^ | ^$^ | ^#^ |
|  | Set 2 - SIT (N.m^-1^) | 2.6 ± 1.4 | 4.5 ± 3.0 | 5.3 ± 2.3 | 7.1 ± 3.1 | 10.8 ± 5.3 | ^*^ | ^$^ | ^#^ |
| **Visit 2**  **Pre** | Set 1 - Voluntary Contractions (N.m^-1^) | 240 ± 49 | 211 ± 43 | 182 ± 39 | 150 ± 30 | 120 ± 25 |  | ^$^ |  |
|  | Set 2 - Voluntary Contractions (N.m^-1^) | 235 ± 50 | 207 ± 46 | 177 ± 39 | 149 ± 31 | 122 ± 27 |  | ^$^ |  |
|  | Set 1 - SIT (N.m^-1^) | 2.0 ± 1.2 | 3.7 ± 2.0 | 6.9 ± 2.6 | 12.7 ± 3.5 | 20.0 ± 4.5 |  | ^$^ | ^#^ |
|  | Set 2 - SIT (N.m^-1^) | 2.0 ± 1.9 | 3.6 ± 2.1 | 6.7 ± 3.7 | 11.4 ± 3.2 | 19.5 ± 3.8 |  | ^$^ | ^#^ |
| **Visit 2** | Set 1 - Voluntary Contractions (N.m^-1^) | 172 ± 30 | 151 ± 26 | 130 ± 21 | 109 ± 16 | 87 ± 14 | ^*^ | ^$^ |  |
| **Post** | Set 2 - Voluntary Contractions (N.m^-1^) | 172 ± 30 | 149 ± 25 | 128 ± 21 | 107 ± 17 | 88 ± 14 | ^*^ | ^$^ |  |
|  | Set 1 - SIT (N.m^-1^) | 3.2 ± 1.7 | 4.8 ± 2.4 | 6.1 ± 2.6 | 9.2 ± 3.1 | 13.5 ± 5.8 | ^*^ | ^$^ | ^#^ |
|  | Set 2 - SIT (N.m^-1^) | 2.8 ± 1.5 | 4.7 ± 2.5 | 7.2 ± 2.4 | 9.7 ± 3.7 | 12.2 ± 4.9 | ^*^ | ^$^ | ^#^ |

^*^ Pre- vs post-fatiguing task difference (P<0.05); ^$^ Significant effect of contraction level (P<0.05); # Significant effect for session (P<0.05)
