# Supplementary material for: Improving the measurement of TMS-assessed voluntary activation in the knee extensors
Source: PLoS One. 2019 Jun 6;14(6):e0216981. doi: 10.1371/journal.pone.0216981 (PMC6553714; doi:10.1371/journal.pone.0216981)
Supplement: S1 Fig — (DOCX) [file pone.0216981.s005.docx]

**S1 Fig: Example of a curvilinear relationship for one participant.**
